# Inhibitory effects of carvacrol on glucansucrase from *Streptococcus mutans* and salivary α-amylase: in silico and in vitro studies

**DOI:** 10.55730/1300-0152.2727

**Published:** 2025-01-08

**Authors:** Samet KOCABAY, M. Abdullah ALAGÖZ, Birnur AKKAYA

**Affiliations:** 1 Department of Molecular Biology and Genetics, Faculty of Science and Literature, İnönü University, Malatya, Turkiye; 2 Department of Pharmaceutical Chemistry, Faculty of Pharmacy, İnönü University, Malatya, Turkiye; 3 Department of Molecular Biology and Genetics, Faculty of Science, Sivas Cumhuriyet University, Sivas, Turkiye

**Keywords:** Carvacrol, glucansucrase, *Streptococcus mutans*, biofilm, amylase

## Abstract

**Background/aim:**

*Streptococcus mutans* produces glucansucrase, an enzyme that converts sucrose into lactic acid, which lowers the pH in the oral environment and leads to tooth enamel demineralization, a key factor in dental caries. Additionally, glucansucrase facilitates the formation of extracellular polysaccharides, which promote bacterial adhesion to tooth surfaces. This study investigates the inhibitory effects of carvacrol, a natural compound, on glucansucrase activity both in vitro and in silico.

**Materials and methods:**

Glucansucrase enzyme was purified from *S. mutans*. The inhibitory effects of carvacrol against glucansucrase enzyme were investigated both in vitro and in silico.

**Results:**

In the presence of 50 mM carvacrol, glucansucrase and salivary amylase activities were reduced by 51.25% and 14.85%, respectively. Carvacrol did not significantly inhibit (4.73%) the salivary amylase enzyme at 10 mM. Glucansucrase activity decreased by 51.63% in the presence of 10 mM acarbose, which was used as a positive control in glucansucrase enzyme studies. Acarbose inhibited salivary amylase with 82.54% loss of enzyme activity in the presence of 1 mM acarbose. The docking score obtained for carvacrol was −5.262 kcal/mol, while that obtained for acarbose was −6.084 kcal/mol. We carried out molecular dynamics simulation studies for 100 ns to determine the stability of carvacrol in the active site of the protein. Carvacrol demonstrated stable binding to glucansucrase with hydrogen bonds and interactions at key residues (ASP477, GLN960, and ASP909), confirmed by molecular dynamics simulations. Carvacrol remained stable between 16 and 100 ns.

**Conclusion:**

Carvacrol selectively inhibits glucansucrase without significantly affecting salivary amylase, making it a more targeted inhibitor compared to acarbose, which inhibits both enzymes. Docking studies indicated that while carvacrol has a lower binding affinity than acarbose, its stable interaction with the enzyme suggests sustained inhibitory action. These findings highlight carvacrol as a promising natural compound for preventing dental caries, offering a more selective alternative to traditional inhibitors. Further in vivo studies are necessary to assess its therapeutic efficacy and safety in clinical applications for oral health.

## 1. Introduction

Dental caries, recognized as one of the most common human diseases among all age groups, is a sugar-dependent multifactorial disease in which *Streptococcus mutans* and other cariogenic species interact with dietary components and trigger virulence (Xu et al., 2011; Ren et al., 2016a; Zhang et al., 2021). The glucansucrase secreted by *S. mutans* and virulence genes alters the oral microbiota and creates a sticky environment for dental biofilm-forming structural bacterial colonies (Nguyen et al., 2014). Glucansucrase, produced by the oral bacterium *S. mutans*, converts sucrose to lactic acid. The released lactic acid lowers the pH value around the teeth. These low pH values are the direct cause of tooth decay as they cause the calcium phosphate in the tooth enamel to dissolve. Furthermore, glucan, an extracellular polysaccharide (Zhang et al., 2021), accumulates as a result of glucansucrase enzyme activity and facilitates the adhesion of *S. mutans* to the surfaces of the teeth. Depending on the polymer density produced, its presence supports the surface adhesion of bacteria that produce more acid (Nasry et al., 2016).

Current practices for preventing dental caries indiscriminately remove bacteria from the mouth chemically and physically, such as the use of mouthwash or toothbrushing (Hailu et al., 2020). The development of selective new inhibitors related to bacterial virulence mechanisms that can target important virulence factors such as glucansucrase without disrupting the structure of the oral microflora or inhibiting other enzymes is now seen as an important scientific strategy (Ren et al., 2016b; Feng et al., 2019). In the literature, small molecules obtained from many different natural sources have been studied to evaluate the antibiofilm activity of the glucosyltransferase enzyme and the inhibition of cariogenic plaque formation by *S. mutans* (Nijampatnam et al., 2018; Yang et al., 2021). Although the potential glucansucrase activity of mangiferin has been studied, its applications are limited due to the fact that it inhibits other enzymes in the saliva (Emeka et al., 2020). Proposed inhibitors should not inhibit other enzymes in the mouth while inhibiting glucansucrase.

**Figure 1:**
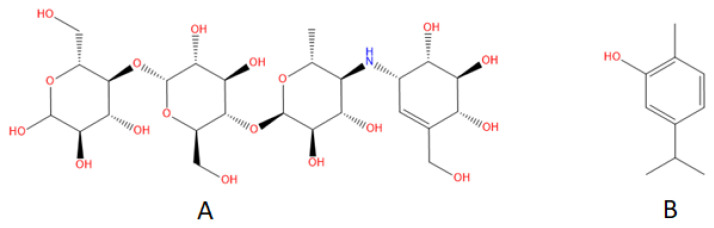
Structures of acarbose **(A)** and carvacrol **(B)** (obtained from https://go.drugbank.com/).

Acarbose (Figure 1A) is a positive inhibitor of glucansucrase enzyme. However, it also inhibits the salivary amylase enzyme. Carvacrol (Figure 1B) has a monoterpenic structure and is found in many plant species, especially among the taxa of *Origanum* and *Thymus*. It exerts antibiofilm activities against *S. mutans* (Khan et al., 2017). Due to its solubility in water and its hydrophobic character, it has the ability to penetrate biofilm layers (Zhang et al., 2018). In the present study, carvacrol was chosen over other glucansucrase inhibitors due to its selectivity, bioavailability, and ability to penetrate biofilms, which are critical factors for an effective and targeted approach to dental caries prevention. Unlike other glucansucrase inhibitors such as acarbose, which can inhibit salivary amylase and disrupt the normal digestive process, carvacrol specifically targets glucansucrase activity without significantly affecting other enzymes in the oral cavity. This selectivity is crucial as it minimizes potential side effects and preserves the delicate balance of the oral microbiome. Furthermore, carvacrol’s ability to penetrate biofilms makes it particularly effective in disrupting the bacterial colonies that form on tooth surfaces, where glucansucrase activity occurs. These distinctive properties give carvacrol a clear advantage over other inhibitors that may not be able to effectively target the biofilm matrix or provide sufficient stability and potency at the site of infection (Amaral et al., 2015).

Computer-assisted strategies have become an indispensable part of targeted drug design (Cortés et al., 2004). With the use of these methods, rapid decision-making can be achieved with various decision-making approaches, such as previewing the active history (virtual activity screening) from large libraries of ligands, identifying derivatives to be derived and synthesized from skeletons of certain sizes, revealing ligand–receptor interactions, or estimating specific pharmacokinetic and toxic waste products in detail (Thomford et al., 2018; Bruno et al., 2019). With increasing knowledge of molecular death, powerful new tools for drug design have been introduced in recent years, and these tools have led to the discovery of various drugs and drug candidates as well as anticancer clinical candidates such as nolatrexed (Islam et al., 2022).

This study highlights carvacrol’s unique ability to selectively inhibit glucansucrase without affecting salivary amylase, making it a novel and targeted approach to dental caries prevention. In contrast to traditional treatments that indiscriminately target a broad range of oral bacteria, this study advances current methodologies by focusing on a specific virulence factor crucial to caries formation, thus preserving the beneficial oral microbiome. This selective inhibition fills a significant gap in the existing research by offering a more precise and sustainable alternative to broad-spectrum antibacterial strategies. With that aim, the inhibitory effects of carvacrol on both salivary amylase and glucansucrase were investigated.

## 2. Materials and methods

Acarbose (95%, Lot 459080010, Thermo Scientific, Waltham, MA, USA), carvacrol (≥97.5%, Lot A0438958, Thermo Scientific), glucansucrase from *S. mutans* and α-amylase from human saliva (type XIII-A, lyophilized powder, A0521, Sigma, St. Louis, MO, USA), sucrose (residue on ignition (ash) of <100 ppm, Carlo Erba, Cornaredo, Italy), soluble starch (residue on ignition (ash) of 0.3%, Merck, Darmstadt, Germany), brain heart infusion (BHI) broth (Merck), and 3,5-dinitrosalicylic acid (DNS; 98%, Tokyo Chemical Industry, Tokyo, Japan) were utilized in this study. Other chemicals not specified here were of analytical grade.

### 2.1. Extracellular glucansucrase production and partial purification

BHI was prepared to include 5% sucrose. The BHI reagent (37 g) was dissolved in 1000 mL of distilled water and autoclaved. *S. mutans* was inoculated on fresh medium overnight at 37 °C and 120 rpm (Emeka et al., 2020). Aliquot samples were read at a wavelength of 600 nm at different intervals during the growth period. The released extracellular bacterial glucansucrase was isolated from the culture medium (Kocabay et al., 2016). Cells were first removed by centrifugation for 15 min at 4000 rpm and 4 °C. The supernatant was mixed with absolute ethanol at a 1:2 (V/V) ratio. The mixed solution was then incubated at −20 °C overnight to precipitate all proteins. The solution was centrifuged for 15 min at 4000 rpm and 4 °C for the collection of precipitated proteins and lyophilized at −60 °C.

### 2.2. Glucansucrase inhibition assay

Glucansucrase inhibition by carvacrol and acarbose (positive control) was analyzed using DNS (Kocabay et al., 2016). Different concentrations of carvacrol and acarbose ranging from 0 to 200 mM were prepared in 0.02 M acetate buffer (pH 5) with 5% sucrose (Emeka et al., 2020). Subsequently, enzyme solutions of 20 mg/mL were prepared in 0.02 M acetate buffer (pH 5), and 200 μL of substrate and 200 μL of enzyme were mixed and incubated for 18 h at 37 °C and 100 rpm. Control samples were prepared without carvacrol and acarbose. The reaction mixture was treated with 400 μL of DNS reagent, which was prepared by dissolving 10 g of DNS and 300 g of sodium potassium tartrate in 800 mL of 0.5 N NaOH solution. The mixture was then gently heated to facilitate the dissolution of the reagents. After complete dissolution, the final volume was adjusted to 1.0 L using distilled water (for the acarbose experiment, 60 μL of substrate and 60 μL of enzyme were mixed and incubated for 18 h at 37 °C and 100 rpm, and then 120 μL of DNS reagent was added to the mixture). The new solution was boiled in water for 5 min and, after cooling, the absorbance values were recorded. Experiments were performed in triplicate. One unit of glucansucrase activity was defined as the amount of enzyme required to generate 1 mg of fructose per minute under optimal conditions, as determined using a fructose standard curve.

To calculate the inhibition percentages of glucansucrase and salivary amylase, the following equation is typically used:


Inhibition percentage=((Absorbance of control group-Absorbance in presence of inhibitor)/Absorbance of control group)×100

Here, “Absorbance of control group” refers to the enzyme activity in the absence of the inhibitor (i.e., baseline enzyme activity), “Absorbance in presence of inhibitor” refers to the enzyme activity after the addition of the inhibitor (in this case, carvacrol at specific concentrations), and the result is multiplied by 100 to express the inhibition as a percentage. This formula can be similarly applied for other concentrations and enzymes to assess their inhibitory effects.

### 2.3. Saliva amylase inhibition assay

Amylase from human saliva was analyzed with carvacrol and acarbose (positive control) (Kandra et al., 2005; Kocabay et al., 2016). Different concentrations of carvacrol and acarbose ranging from 0 to 200 mM were prepared in 0.02 M phosphate buffer (pH 7.0) with 5% starch. Enzyme solution of 0.02 mg/mL was prepared in 0.02 M phosphate buffer (pH 7.0), and 200 μL of substrate and 200 μL of enzyme were mixed and incubated for 24 h at 37 °C and 100 rpm. Control samples were prepared without carvacrol and acarbose. After the incubation period, 400 μL of DNS reagent was added to the mixture. The resulting solution was boiled in water for 5 min. After cooling, samples were diluted ten times and then absorbance values were recorded. Experiments were performed in triplicate. One unit of amylase activity was defined as the quantity of enzyme needed to produce 1 mg of maltose per minute under optimal conditions, as determined by the maltose standard curve.

### 2.4. Docking studies

Maestro 12.8 software (Schrödinger Release 2021-4, Schrödinger LLC, New York, NY, USA) was used in all docking studies. The molecular docking protocol was performed systematically as follows: For ligand preparation, 2D Sketcher was used to draw the ligand structures and LigPrep (Schrödinger LLC) was utilized to minimize the ligands and generate 3D conformers. A pH value of 7.0 ± 2.0 was set to predict protonation states (Madhavi Sastry et al., 2013). For protein preparation, the X-ray structure of the target protein (PDB ID: 3AIC) was retrieved from the RCSB Protein Data Bank (www.rcsb.org) (Madhavi Sastry et al., 2013; Peele et al., 2020). Schrödinger’s Protein Preparation Wizard, Prime, Impact, Epic, and PropKa modules were used to preprocess the protein, including the removal of water molecules, insertion of missing hydrogen atoms, assigning of charges, and optimization of the protein structure at physiological pH (Olsson et al., 2011). For grid generation, a grid map centered on the active site of the protein was generated using the Glide Grid Generation module in Maestro. The grid box dimensions were set to include all key residues within a 10-Å radius around the active site (Algul et al., 2021). For the docking procedure, molecular docking was performed using the Glide module in standard precision mode. Each ligand was docked into the prepared grid 100 times to explore possible binding poses. Docking results were ranked based on the GlideScore, which evaluates binding affinities by considering molecular mechanics and solvation energies (Friesner et al., 2004). Finally, for docking analysis, the interactions of the ligands with the residues in the target region of the protein (polar, hydrophobic, hydrogen bonding, etc.) were examined at the end of the docking studies with Maestro’s pose viewer.

### 2.5. Molecular dynamics simulations

Molecular dynamics simulations were carried out according to the parameters given in the Table (Schrödinger Release 2021-4: Desmond Molecular Dynamics System, D. E. Shaw Research, New York, NY, USA; Maestro-Desmond Interoperability Tools, Schrödinger, New York, NY, USA) (Bowers et al., 2006) for 100 ns (Vieira et al., 2024) with Schrödinger’s Maestro package to view the stability and interaction profiles of ligand–protein complexes. The average distances between the backbone atoms of the protein–ligand structures, referred to as backbone root mean square deviations (RMSDs), were plotted for the comparison of dynamic and structural properties (Ozten et al., 2021).

## 3. Results and discussion

### 3.1. Bacterial growth and enzyme production

**Figure 2:**
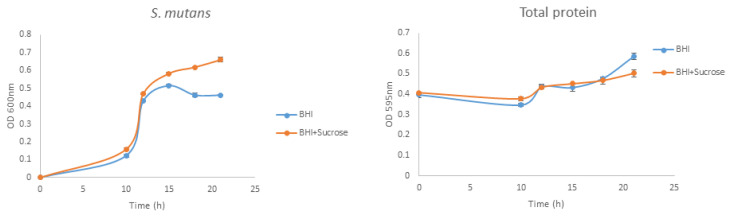
Bacterial growth and enzyme production versus time.

The growth of *S. mutans* and protein production results of the bacteria against time when inoculated on BHI broth medium with or without 1% sucrose are shown in Figure 2. After 10 h of inoculation, the bacterial growth reflected a logarithmic phase. Total protein production resembled the growth curve.

### 3.2. Glucansucrase inhibition

**Figure 3:**
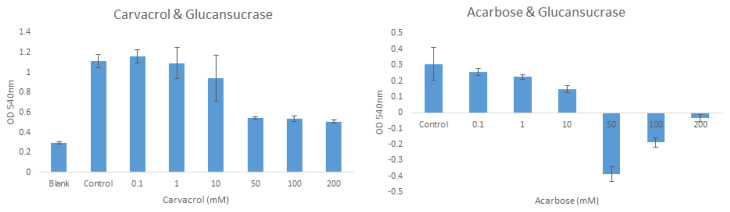
Inhibitory effects of carvacrol and acarbose on purified glucansucrase.

The inhibition of glucansucrase by carvacrol and acarbose molecules was evaluated at different concentrations. According to the results for carvacrol, the optical density (OD) values were 0.294, 1.114, 1.163, 1.094, 0.942, 0.543, 0.536, and 0.507 for the blank, the control (0), and 0.1, 1, 10, 50, 100, and 200 mM, respectively. For acarbose, the OD values were 0.306, 0.257, 0.225, 0.148, −0.384, −0.187, and −0.033 for the control (0) and 0.1, 1, 10, 50, 100, and 200 mM, respectively (Figure 3).

*S. mutans* is the primary bacterium of the oral microbiota that causes dental caries. The biofilm structure produced by *S. mutans* is related to the activity of the glucansucrase enzyme of the bacterium. Although scientists recommend a large number of molecules for inhibition studies of enzyme activities, that is not possible in the event of the inhibition of salivary amylase in the mouth. Therefore, molecules that will inhibit the glucansucrase enzyme while not inhibiting salivary amylase constitute the best candidates. In this study, the potential inhibitory activity of carvacrol against glucansucrase was investigated. Compared to the control group, the enzyme activity started to decrease (15.43%) due to the presence of 10 mM carvacrol. In the presence of 50 mM carvacrol, enzyme activity was reduced by 51.25%. A previous study reported that epigallocatechin gallate (EGCG) reduced the activity of glucansucrase by 45% at a concentration of 25 μg/mL and by 91% at 400 μg/mL (Islam et al., 2020). In another study, it was observed that mangiferin had a 75% inhibitory effect at a concentration of 500 μM against the glucansucrase enzyme isolated from *Streptococcus mutans* (Emeka et al., 2020). While EGCG demonstrates the strongest inhibition at higher concentrations, carvacrol stands out as a promising inhibitor with a more moderate yet selective inhibitory effect. It achieves this effect without significantly affecting salivary amylase, making it a potentially advantageous candidate for oral health applications as it avoids the side effects of inhibiting important digestive enzymes. Therefore, while EGCG and mangiferin are effective inhibitors, carvacrol may offer a more balanced approach in terms of enzyme specificity and potential therapeutic applications for preventing dental caries. However, the practical significance of this inhibition should be evaluated in relation to clinically relevant thresholds. In comparison to acarbose, a known glucansucrase inhibitor, carvacrol’s selectivity is advantageous, as acarbose inhibits both glucansucrase and salivary amylase, potentially disrupting normal digestive processes. Carvacrol, by selectively targeting glucansucrase without significantly affecting amylase at lower concentrations, may offer a more focused and safer approach for oral health applications. While the 50 mM dose showed promising in vitro results, further investigations are needed to determine whether such a concentration can be safely achieved in vivo, especially within the oral cavity. It is likely that much lower doses would be required for effective local concentrations without toxicity. Thus, carvacrol holds potential as an alternative to broad-spectrum inhibitors, but its efficacy and safety at lower concentrations need thorough evaluation.

### 3.3. Saliva amylase inhibition

**Figure 4:**
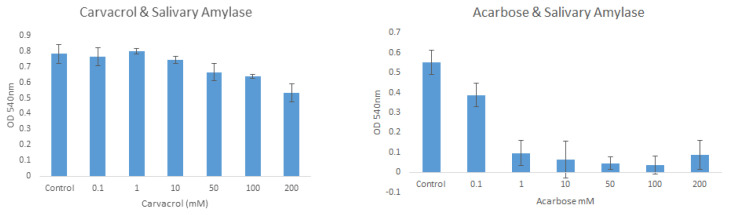
Inhibitory effects of carvacrol and acarbose on salivary amylase.

The inhibition of human salivary amylase by carvacrol and acarbose molecules was studied at different concentrations. According to the results for carvacrol, the OD values were 0.781, 0.762, 0.798, 0.744, 0.665, 0.638, and 0.533 for the control (0) and 0.1, 1, 10, 50, 100, and 200 mM, respectively. For acarbose, the OD values were 0.550, 0.387, 0.096, 0.064, 0.045, 0.037, and 0.086 for the control (0) and 0.1, 1, 10, 50, 100, and 200 mM, respectively (Figure 4).

Based on the results of the salivary amylase enzyme inhibition studies performed for carvacrol, the activity loss was 4.73%, 14.85%, and 31.75% at 10 mM, 50 mM, and 200 mM, respectively. Further amylase enzyme inhibition studies of carvacrol are available in the literature (Aazza et al., 2016).

The acarbose molecule, known to be a glucansucrase enzyme inhibitor, experienced activity loss of 51.63% in the presence of 10 mM acarbose compared to the control group. In the presence of acarbose at 50 mM or more, the activity was measured with negative values. This confirms the inhibitory effect of acarbose against glucansucrase. A previous study reported 70% glucosyltransferase enzyme inhibition in the presence of 0.5 mM acarbose (Hartman et al., 2021).

When the results for the amylase enzyme and acarbose were evaluated, enzyme activity was found to be decreased by 82.54% in the presence of 1 mM acarbose. This result occurred in parallel with the increasing amount of acarbose. Previous studies confirmed that acarbose has a strong inhibitory effect on human salivary amylase (Yoon and Robyt, 2003; Kandra et al., 2005).

Carvacrol’s selective inhibition of glucansucrase and its minimal impact on salivary amylase position it as a promising active ingredient for oral care products, offering a targeted approach to dental caries prevention. Its antibiofilm and antiadhesion properties make it suitable for inclusion in toothpaste, mouthwashes, and oral gels. Carvacrol can inhibit glucansucrase without disrupting normal digestive enzymes, providing an alternative to broad-spectrum inhibitors. Its ability to penetrate biofilms and reduce the adhesion of *S. mutans* enhances its effectiveness in preventing plaque formation. Clinical studies are necessary to assess carvacrol’s in vivo efficacy, bioavailability, safety, and potential toxicity. Furthermore, clinical trials evaluating it in combinations with other agents like fluoride or xylitol could enhance its preventive capabilities. Regulatory approval will be essential for incorporating carvacrol into commercial dental products, ensuring that it is safe, effective, and stable over time.

The fact that acarbose is an amylase inhibitor makes carvacrol a good candidate for use as a glucansucrase enzyme inhibitor. These results were supported by molecular modeling and dynamic simulation studies.

### 3.4. Docking studies

**Figure 5:**
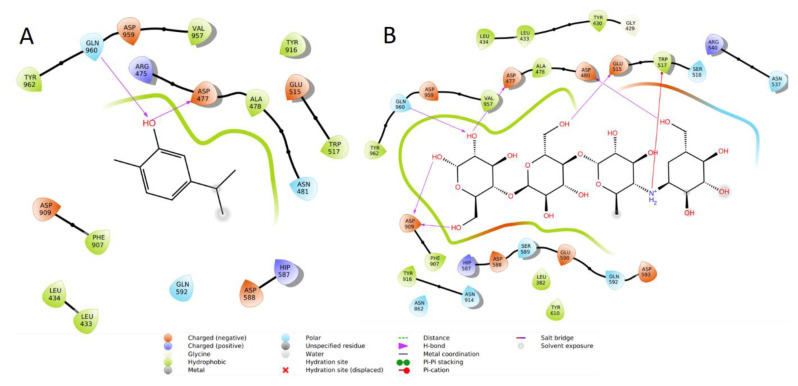
The 2D binding modes of carvacrol and acarbose in the active site.

Docking studies were carried out to determine the binding modes of the protein to the active sites of carvacrol and acarbose as the reference compound. In modeling studies, the docking score of carvacrol was −5.262 kcal/mol, while that of acarbose was calculated as −6.084 kcal/mol. In modeling studies in the literature conducted with 3AIC, it was reported that LEU333, TYR430, ASP477, ALA478, GLU515, ASP588, GLN960, and ASP959 are key residues, and the modeling scores of compounds interacting with these residues are high (Nijampatnam et al., 2018). When the binding modes of the ligands of interest were examined in this study, it was seen that carvacrol formed hydrogen bonds with key residues ASP477 and GLN960. Carvacrol also had polar interactions with GLN960, GLN592, and ASN481; hydrophobic interactions with LEU434, ALA478, TRP517, PHE907, TYR916, VAL957, and TYR962; charged (negative) interactions with ASP477, ASP588, GLU515, ASP909, and ASP959; and charged (positive) interactions with HIS587 and ARG475 (Figure 5A).

Acarbose formed hydrogen bonds with ASP477, ASP909, ASP480, GLU515, and ASP909. It had polar interactions with ASN914, ASN862, SER518, and ASN537; hydrophobic interactions with TYR916, TYR610, PHE907, ALA478, LEU382, LEU430, LEU433, LEU434, and TRP517; and charged (negative) interactions with HIS587 and ARG475. It can be said that these two compounds have similar interactions in general. Like carvacrol, acarbose formed hydrogen bonds with ASP909 and ASP477. Unlike carvacrol, acarbose formed hydrogen bonds with ASP480, GLU515, and ASP909 (Figure 5B). For the validation of the molecular docking studies, the acarbose (as the native ligand) in the 3AIC crystal structure was removed, minimized, and redocked. The RMSD value of the redocked result was calculated as approximately 2 Å. The results of the molecular dynamics simulations also supported the validation of the method. The average RMSD value of 3 Å obtained in the molecular dynamics simulation confirmed that carvacrol bound to the target protein and remained stable.

### 3.5. Molecular dynamics simulation

**Figure 6:**
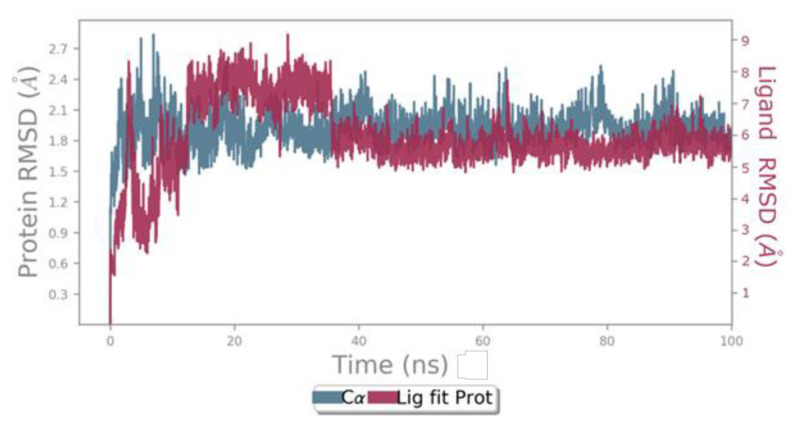
Molecular dynamics simulation process for carvacrol 3AIC. Blue: Cα (RMSD evolution of the protein). Pink: Lig Fit Prot (RMSD of the ligand in the case of the protein–ligand complex).

**Figure 7:**
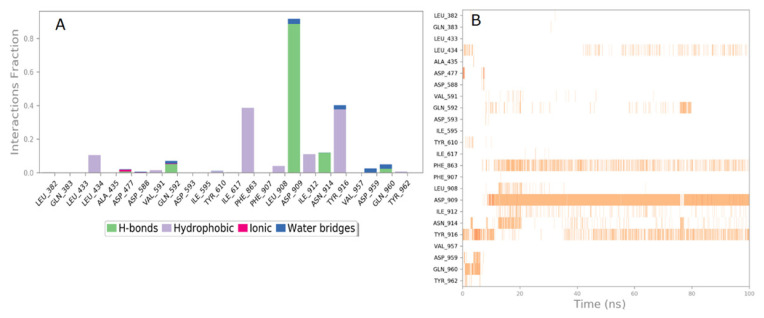
**(A)** Fraction of residue interactions obtained during molecular dynamics stimulation with carvacrol in 3AIC. **(B)** Interactions of carvacrol with the residues in each trajectory frame in 3AIC.

Dynamic simulation studies were carried out to support the molecular modeling studies. Simulation of the ligands at the active site of the protein continued for 100 ns (Figure 6). The RMSD values for Cα ranged between 1.2 and 2.7 Å during the simulations. The RMSD value of carvacrol in the first 16 s varied between 2 and 6 Å. After 16 ns, it changed its conformation, moving away from TYR916 and approaching ASP909 and PHE863 (Figure 7). Subsequently, the RMSD value was observed to range between 5.5 and 8.5 Å, and from 36 ns onwards, the graph roughly reached plateau values and was nearly linear. Carvacrol did not interact with ASP909, one of the important residues in the active region, from the beginning of the simulation. After 16 ns, carvacrol started to form hydrogen bonds with ASP909 and started to interact with PHE863. The hydrophobic interaction with TYR916 also began again after 36 ns. The interactions between carvacrol and ASP909, TYR916, and PHE863 continued throughout the simulation. The presence of hydrogen bonding with ASP909 continued for approximately 88% of the simulation period (Figure 7A). During the simulation process, it was observed that interactions with ASP909, TYR916, and PHE863 were effective in the conformational changes of carvacrol at 16 and 36 ns (Figure 7B).

### 3.6. Conclusions

This study has highlighted the potential of carvacrol as a selective inhibitor of glucansucrase, a key enzyme involved in the development of dental caries. Carvacrol’s selective inhibition of glucansucrase, compared to acarbose, offers a targeted and safer alternative for oral health applications. Unlike acarbose, which also inhibits salivary amylase and could interfere with normal digestive processes, carvacrol minimally impacts amylase, preserving essential digestive functions. This specificity is crucial for oral care, as it ensures that carvacrol can inhibit caries formation without disrupting the balance of enzymes necessary for digestion and overall oral health.

Molecular docking (docking score: −5.262 kcal/mol) and dynamics simulations (average RMSD: 3 Å from 36 ns to the end of the simulation) provided further support for carvacrol’s potential as an effective glucansucrase inhibitor. The stable interaction between carvacrol and the enzyme suggests that carvacrol binds effectively to the enzyme’s active site. Moreover, molecular dynamics simulations confirmed that carvacrol remained stable in the active site throughout the simulation period, providing strong evidence for its therapeutic potential. These findings suggest that carvacrol could serve as a highly effective and specific agent for preventing dental caries by targeting glucansucrase without negatively affecting other important enzymatic functions in the oral cavity.

The docking score of carvacrol was −5.262 kcal/mol, while that of acarbose was −6.084 kcal/mol. The OD values obtained for carvacrol were 1.163, 1.094, and 0.942 and the OD values of acarbose were 0.257, 0.225, and 0.148 at 0.1, 1, and 10 nM. We discovered that acarbose, a glucansucrase inhibitor, had both a higher docking score and higher enzyme inhibition values than carvacrol at different concentrations. These results suggest that the computational and experimental results support each other.

Carvacrol’s unique ability to penetrate biofilms due to its hydrophobic nature further enhances its potential for use as an active ingredient in dental products such as toothpaste, mouthwashes, and oral gels. These formulations could offer a more targeted approach to caries prevention by inhibiting glucansucrase and preventing biofilm formation while also preserving the natural balance of the oral microbiome. This stands in contrast to traditional inhibitors, which often have broader enzyme activities and can disrupt the delicate balance of beneficial oral bacteria. By incorporating carvacrol into oral care products, manufacturers could offer a more selective, effective, and safe option for individuals at high risk of developing dental caries. Furthermore, carvacrol’s selective inhibition of glucansucrase makes it particularly beneficial for individuals prone to dental caries due to an overgrowth of *Streptococcus mutans*. For these individuals, carvacrol-based oral care products could provide a personalized solution for caries prevention without compromising overall oral health.

However, there are several important limitations that must be addressed. First, this study did not include toxicity assessments, which are crucial for evaluating the long-term safety of carvacrol in oral health applications. Without such studies, it is impossible to fully determine whether carvacrol could cause cytotoxicity or irritation in oral tissues, which would be critical factors in its use in commercial oral care products. Additionally, this study has primarily focused on glucansucrase and amylase inhibition; it did not assess carvacrol’s effects on other relevant enzymes involved in oral health, such as glucosyltransferases or other enzymes associated with the oral microbiota. Exploring the broader enzyme specificity of carvacrol is important to understand its selectivity and identify any potential off-target effects that may impact its overall efficacy or safety.

To fully translate these promising results into real-world applications, clinical studies are necessary. These studies should assess the optimal concentration of carvacrol in oral care products, ensuring both efficacy and safety. It is also important to investigate the potential for combination therapies, wherein carvacrol would be used alongside other preventive agents such as fluoride or xylitol, to enhance overall oral health. Clinical trials should also explore the effectiveness of carvacrol among different populations, such as children or individuals with compromised immune systems, to determine its versatility as a preventive treatment.

From a regulatory perspective, the incorporation of carvacrol into commercial oral care products will require rigorous safety and efficacy testing. Regulatory approval will be needed to ensure that carvacrol does not cause irritation or other adverse effects when used in daily oral care routines. Additionally, formulation stability and shelf-life need to be evaluated to ensure that carvacrol retains its therapeutic properties over time.

Carvacrol’s selective inhibition of glucansucrase and its minimal impact on salivary amylase position it as a promising new agent for the prevention of dental caries. The findings of this study suggest that carvacrol could offer a more targeted and safer approach to oral care compared to traditional inhibitors like acarbose. However, further research is required to address the gaps in our understanding, particularly regarding toxicity, enzyme specificity, and in vivo efficacy. By addressing these limitations through comprehensive clinical studies, toxicity assessments, and broader enzyme profiling, carvacrol could emerge as a valuable and effective agent for preventing dental caries, ultimately providing a new focused solution for oral health care.

## Figures and Tables

**Table:** Program parameters of the molecular dynamics stimulation studies.

Force field	OPLS3E (Harder et al., 2016)
Solvation	Crystallographic water (TIP3P)
Counterions	Na^+^, Cl^−^
Ensemble	Nose-Hoover thermostat, 300k barostat, 1 bar
Boundary conditions	Orthorhombic periodic boundary conditions
Buffer region	10 Å
Deleted molecules	Water, etc.
Minimization algorithm	1000 steps of steepest descent followed by conjugate gradient
Adjusting the concentration of the system	0.15 M NaCl
